# Clinical efficacy of Apatinib combined with Epidermal growth factor receptor - tyrosine kinase inhibitor (EGFR-TKI) in nonsmall cell lung cancer after EGFR-TKI resistance

**DOI:** 10.12669/pjms.40.8.9682

**Published:** 2024-09

**Authors:** Ruifen Tian, Yi Guo, Xing Zhang, Xia Zhang, Xia Song

**Affiliations:** 1 Ruifen Tian, Department of Respiratory Medicine, Shanxi Tumour Hospital, 3 Zhigongxin Village, Taiyuan, Shanxi Province 030013, P.R. China; 2 Yi Guo, Department of Respiratory Medicine, Shanxi Tumour Hospital, 3 Zhigongxin Village, Taiyuan, Shanxi Province 030013, P.R. China; 3 Xing Zhang, Department of Respiratory Medicine, Shanxi Tumour Hospital, 3 Zhigongxin Village, Taiyuan, Shanxi Province 030013, P.R. China; 4 Xia Zhang, Department of Respiratory Medicine, Shanxi Tumour Hospital, 3 Zhigongxin Village, Taiyuan, Shanxi Province 030013, P.R. China; 5 Xia Song, Department of Respiratory Medicine, Shanxi Tumour Hospital, 3 Zhigongxin Village, Taiyuan, Shanxi Province 030013, P.R. China

**Keywords:** Apatinib, Epidermal growth factor receptor, Tyrosine kinase inhibitor, Non-small cell lung cancer, Resistance

## Abstract

**Objective:**

To evaluate the efficacy and safety of Apatinib combined with epidermal growth factor receptor - tyrosine kinase inhibitor (EGFR-TKI) in the treatment of patients with non-small cell lung cancer (NSCLC) and acquired EGFR-TKI resistance.

**Methods:**

Clinical records of 106 patients with NSCLC at Shanxi Tumor Hospital of the Chinese Academy of Medical Sciences Cancer Hospital from January 2017 to October 2020, with acquired drug resistance after EGFR-TKI treatment were retrospectively analyzed. Among them, 52 patients received Apatinib combined with EGFR-TKI (Apatinib group), and 54 patients received a standard chemotherapy (pemetrexed combined with platinum) (chemotherapy group). Clinical efficacy indicators, follow-up results, and adverse reactions in both groups were compared.

**Results:**

There was no significant difference in the objective response rate and disease control rate between the two groups (*P*>0.05). The progression free survival (PFS) of the Apatinib group was significantly longer than that of the chemotherapy group (10.5 months vs. 5.7 months; *P*<0.05). There was no significant difference in adverse reactions between the two groups (*P*>0.05).

**Conclusions:**

Compared with standard chemotherapy, Apatinib combined with EGFR-TKI has the same efficacy in treating NSCLC patients with EGFR-TKI resistance, and was associated with longer PFS with no significant increase in adverse reactions.

## INTRODUCTION

Lung cancer (LC) is the most common malignancy worldwide, and non-small cell lung cancer (NSCLC) accounts for approximately 80% of all lung cancer cases.^1^ In China, LC mortality rate has increased by 465% in the last 30 years.^2^ It has been reported that 57% of lung cancer patients present with distant metastasis at initial diagnosis.^3^ Therefore, treatment of patients with advanced stages of the disease is an important component of the lung cancer treatment system.

In recent years, multiple Phase-III clinical studies (IPASS, NEJGSG, WJTOG3405, First Signal, Optimal, etc.) have shown that tyrosine kinase inhibitor (TKI), such as gefitinib or erlotinib, are effective as first-line treatment in patients with epidermal growth factor receptor (EGFR) mutation, and are associated with good response rate, longer progression free survival (PFS), and better quality of life compared to standard chemotherapy.^4^,^5^ Therefore, EGFR-TKI has been approved for first-line treatment in NSCLC patients with positive EGFR gene mutations. Unfortunately, the efficiency of EGFR-TKI therapy is limited by the drug resistance that develops after an average of 10-12 months of EGFR-TKI treatment. The mechanism of TKI action includes reversible inhibition of the *EGFR-TK* domain by competitively binding to adenosine triphosphate (ATP).^6^ It is shown that in about 50% of the cases, EGFR-TKI resistance is due to the substitution of threonine at the 790 positions of EGFR20 exon with methionine (T790m), which alters the affinity of the inhibitor to ATP and prevents EGFR-TKI from effectively blocking EGFR signaling pathway, resulting in resistance.^7^

Development of tumor neovascularization involves secretion of various vascular- growth factors by rapidly growing tumor cells under hypoxic conditions, thus stimulating tumor angiogenesis.^8^ One of the important growth factors is Vascular Endothelial Growth Factor (VEGF) that binds to the VEGF receptor (VEGFR), stimulating downstream signal transduction and ultimately leading to tumor angiogenesis.^9^ The theoretical basis for the combined application of VEGF and EGFR-TKI suggests that there is a common downstream signaling pathway between VEGF and HER-1/EGFR. Therefore, EGFR-TKI can also downregulate VEGF, while VEGF inhibitors can inhibit HER-1/EGFR signaling.^10^ Therefore, it is possible that EGFR-TKI combined with VEGF may not only inhibit cell proliferation and promote cell apoptosis, but also reduce tumor neovascularization and delay the development of the EGFR-TKI resistance.

Apatinib is a small-molecule VEGFR tyrosine kinase inhibitor. In vivo and in vitro experiments have shown that Apatinib has good tumor growth inhibitory activity on lung cancer.^11^ A randomized double blind, placebo parallel controlled Phase-II trial reported that Apatinib may be effectively used as a third (or above) line of treatment for advanced non-small cell lung cancer patients. Compared with the placebo group, median PFS (mPFS) of patients, treated with Apatinib was extended by 2.8 months.^11^,^12^ There are few studies exploring the clinical efficacy of Apatinib combined with EGFR-TKI in NSCLC after EGFR-TKI resistance.^13^ Therefore, this study aimed to assess the efficacy and safety of Apatinib combined with EGFR-TKI for treating NSCLC patients with EGFR-TKI resistance by comparing with chemotherapy.

## METHODS

Clinical records of 106 patients with NSCLC (33 males and 73 females) with acquired drug resistance after EGFR-TKI treatment, treated at Shanxi Tumor Hospital of the Chinese Academy of Medical Sciences Cancer Hospital from January 2017 to October 2020, were retrospectively analyzed. Age of the patients ranged from 29 to 75 years, with an average of 56.07 ± 10.67 years old. Of 106 patients, 52 received Apatinib combined with EGFR-TKI, and 54 received chemotherapy (pemetrexed combined with platinum)

### Ethical Approval:

The present study was conducted according to the Declaration of Helsinki and Ethical Guidelines for Medical and Biological Research Involving Human Subjects. The Ethics Committee of Shanxi Tumour Hospital approved this study (No. 201721) on May 24^th^, 2017. Due to the retrospective nature, written informed consent was not required.

### Inclusion criteria:

- Age 18 and above.
- Late stage (IIIB, IIIC, and IV) non-small cell lung cancer confirmed by pathology, with measurable lesions (tumor lesion CT scan length ≥ 10 mm, lymph node lesion CT scan short diameter ≥ 15 mm, scan layer thickness not greater than 5 mm);
- Patients who have been treated with EGFR-TKI (including erlotinib, gefitinib, and exetinib) and acquired drug resistance, as indicated by the progression of the condition within six months after the last immunotherapy.

### Exclusion criteria:

- Patients with persistent clinical treatment related toxicity associated with previous chemotherapy and/or radiation therapy;
- Patients with active brain metastasis or leptomeningeal disease;
- Patients with radiographic evidence indicating the presence of cavernous or necrotic tumors;
- Patients with radiographic evidence (CT or MRI) indicating the presence of central type tumors invading local large blood vessels;
- Patients who were receiving anticoagulant or antiplatelet therapy;
- Patients with abnormal coagulation function and a tendency to bleed;
- Patients with hypertension who cannot be reduced to normal levels after treatment with antihypertensive drugs (systolic blood pressure >140 mmHg, diastolic blood pressure >90 mmHg).

### Treatment regimens:

EGFR-TKI [Gifitinib (AstraZeneca Pharmaceutical Co., Ltd.; approval No. J20180014; specification: 0.25g), orally, 0.25g/dose, once a day. Ecetinib Hydrochloride Tablets (Beida Pharmaceutical Co., Ltd.; approval No.: H20110061; specification: 125 mg), orally, 125 mg/dose, three times per day. At the same time, Apatinib mesylate tablets (Jiangsu Hengrui Pharmaceutical Co., Ltd.; approval No.: H20140103; specification: 250mg) were added at the initial dose of 500 mg each time, orally 0.5 hours after breakfast, once a day. A treatment cycle was 28 days.

### Chemotherapy:

Vinorelbine tartrate injection (Jiangsu Haosen Pharmaceutical Co., Ltd.; Approval No.: H199900278), 25 mg/m^2^ on the 1st and 8th days; On the 1st to 4th day of chemotherapy, 75 mg/m^2^ of cisplatin (Qilu Pharmaceutical Co., Ltd.; Approval number: H37021356) administered intravenously. Treatment period is 21 days per cycle.

### Efficacy evaluation criteria:

Efficacy was evaluated according to RECIST 1.1. (https://recist.eortc.org/recist-1-1-2/) and classified as complete response (CR), partial response (PR), stable disease (SD), and progressive disease (PD). Objective response rate (ORR)=(CR+PR) cases/total cases × 100%; Disease control rate (DCR)=(CR+PR+SD) cases/total cases × 100%.

*Adverse reactions:* Adverse reactions were evaluated according to the Common Terminology Criteria adverse events (NCI-CTCAE v4.0).^14^

### Statistical analysis:

SPSS 26.0 system software was used for data analysis. Normality of data was evaluated using the Shapiro-Wilk test. Non-normally distributed data were presented as median and interquartile intervals and analyzed using Wilcoxon test. The counting data were expressed in terms of cases or percentages for testing. PFS was tested and analyzed using Kaplan Meier. All statistical tests were conducted using a bilateral test. A *P*-value less than 0.05 was considered statistically significant, with a 95% confidence interval (CI).

## RESULTS

Based on the clinical records, a total of 106 patients met the inclusion criteria. Of them, 52 patients received Apatinib combined with EGFR-TKI, and 54 patients received chemotherapy (Vinorelbine Tartrate Injection). There was no statistically significant difference in baseline data between the two groups of patients (*P*>0.05) (Table-I).

**Table-I:** Comparison of baseline data between two groups of patients.

Group	n	Gender (male/female)	Age (years)	EGFR mutation (Yes)	T70m mutation (Yes)	Brain metastasis (Yes)
Apatinib group	52	20/32	60 (49-63)	33 (63.5%)	7 (13.5%)	14 (26.9)
Chemotherapy group	54	13/41	54 (49-65)	31 (48.4%)	12 (22.2%)	24 (44.4)
/*Z*		2.558	-1.402	0.406	1.382	3.536
P		0.110	0.161	0.524	0.240	0.060

**Fig.1:**
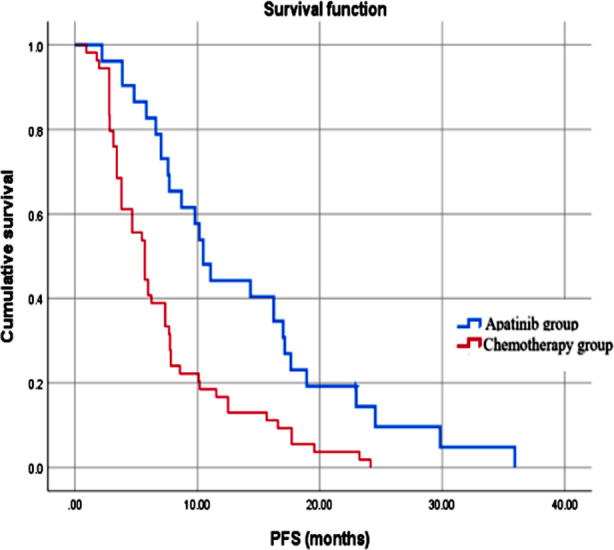
Progression-free survival in the two groups of patients.

There was no significant difference in the objective response rate and disease control rate between the two groups (P>0.05) (Table-II). As shown in Fig.1, the median PFS of the Apatinib group was significantly higher (10.5 months), compared to the PFS of the chemotherapy group (5.7 months; *P*<0.05). There was no statistically significant difference in the incidence of adverse reactions between the two groups (*P*>0.05) (Table-III).

**Table-II:** Comparison of objective response rates and disease control rates between two groups.

Group	n	CR	PR	SD	PD	ORR (%)	DCR (%)
Apatinib group	52	0	11	27	14	21.2	73.1
Chemotherapy group	54	0	15	18	21	27.8	61.1
						1.074	1.715
P						0.300	0.190

**Table-III:** Comparison of adverse reaction rates between two groups.

Adverse event	Apatinib group	Chemotherapy group	χ^2^	P
Diarrhea	33	30	0.687	0.407
Hypertension	33	27	1.954	0.162
Hand-foot skin reaction	21	25	0.377	0.539
Feeble	15	12	0.612	0.434
Proteinuria	13	11	0.324	0.569
Loss of appetite	10	14	1.790	0.181
Elevated total bilirubin	8	9	0.118	0.731
Thrombocytopenia	6	8	0.248	0.618
Elevated transaminase	6	9	0.573	0.449
Vomit	4	5	0.084	0.772
Bleeding	4	6	0.362	0.547

## DISCUSSION

The results of this study showed that there was no significant difference in the objective response rate and disease control rate between patients treated with Apatinib combined with EGFR-TKI and standard chemotherapy. However, combined Apatinib/EGFR-TKI was associated with significantly longer PFS and no significant increase in adverse reactions.

Our results are in agreement with the previous results.^15^,^16^ A study by Li et al.^15^ indicated that using Apatinib for EGFR-TKI re-stimulation can significantly improve PFS. These results suggest that this combination may be a new treatment choice for NSCLC patients with T790m or other type of drug resistance. Similarly, Yu et al.^16^ treated 29 patients with advanced NSCLC who slowly progressed after first-generation EGFR-TKI monotherapy with the combination of original drug and Apatinib, and showed that the ORR, DCR, and mPFS of patients significantly improved, while the rate and the severity of the adverse reactions was acceptable and controllable.

The discovery of EGFR mutations and the emergence of EGFR-TKI have greatly changed the therapeutic prospects of NSCLC, shifting from traditional chemotherapy to molecular targeted therapy.^17^ EGFR-TKI has the characteristics of high efficiency and low toxicity, and has become the first line treatment standard for patients with EGFR mutation.^18^ However, drug resistance is an inevitable issue in EGFR-TKI, especially after third-generation EGFR-TKI resistance, subsequent treatment methods are very limited.^19^ Therefore, there is an urgent need for new treatment strategies to further improve the prognosis of NSCLC patients with EGFR mutation. There is a phenomenon of insufficient blood supply inside the tumor, which is prone to forming a relatively hypoxic area that promotes the survival of cancer cells after systematic combination therapy. Anti-angiogenic drugs stimulate the normalization of tumor blood vessels and improve the local microenvironment of tumors.^20^ The improvement of hypoxia is particularly beneficial for drug delivery, thus reducing chemotherapy resistance and potentially prolonging survival time. In addition, it is plausible that tumor blood vessels in the tumor microenvironment are exposed to interaction with various immune cells. Normalization of blood vessels can reshape tumor microenvironment, and lead to transition from an immunosuppressive to an immune-promoting state, thus improving treatment effectiveness and survival of the patient.^21^

Our results indicated that the most common adverse reactions in patients receiving Apatinib combined with EGFR-TKI treatment are diarrhea and hypertension. This may be due to the unreasonable and excessive use of Apatinib.^22^ Zhao et al.^23^ also pointed out that the most common adverse reaction after using Apatinib is hypertension, while other common adverse reactions are fatigue, proteinuria, etc. While Apatinib is an anti-tumor drug that can block DNA synthesis and prevent normal growth of cancer cells, it can also have similar impact on the DNA synthesis in normal cells.^19^,^22^ Therefore, it is necessary to closely monitor the patient’s basic vital signs, such as blood pressure and urinary protein levels. Our results further strengthen the need for preventive and response measures, such as checking relevant medical history of each patient, adjusting the frequency of Apatinib administration for management, and administering a small amount of Apatinib after the adverse reactions have alleviated.^19^-^21^

### Limitations:

This is a retrospective study with a small number of cases, and there may be selection bias in the sample size. Further expansion of sample size and prospective studies are needed to investigate the efficacy of Apatinib combined with EGFR-TKI in the treatment of acquired drug resistance in patients with NSCLC.

## CONCLUSION

Our study shows that the combination of Apatinib and EGFR-TKIs has good efficacy in treating NSCLC patients with EGFR-TKI resistance, significantly improving their PFS. Adverse reactions, associated with the combined treatment, are controllable and within an acceptable range.

### Authors’ contributions:

**RT** and **YG** conceived and designed the study.

**XZ, Xia Zhang** and **XS** collected the data and performed the analysis.

**RT** and **YG** were involved in the writing of the manuscript and are responsible for the integrity of the study.

All authors have read and approved the final manuscript.
